# Supplementary material for: Autonomic nervous system modulation by G protein-biased mu-opioid receptor agonists: A translational scoping review protocol
Source: PLoS One. 2026 May 15;21(5):e0349596. doi: 10.1371/journal.pone.0349596 (PMC13178854; doi:10.1371/journal.pone.0349596)
Supplement: S1 Checklist — (DOCX) [file pone.0349596.s001.docx]

**PRISMA-P 2015 Checklist**

| **Section/topic** | **#** | **Checklist item** | **Information reported**  **(Yes/No/N/A)** | **Page/Section number**  **(s)** |
| --- | --- | --- | --- | --- |
| **ADMINISTRATIVE INFORMATION** | | | | |
| **Title** |  |  |  |  |
| Identification | 1a | Identify the report as a protocol of a systematic review. | Yes | Page 1 (Title Page); Page 2 (Abstract) - Identified as scoping review protocol |
| Update | 1b | If the protocol is for an update of a previous systematic review, identify as such. | N/A | N/A - Initial protocol |
| Registration | 2 | If registered, provide the name of the registry (e.g., PROSPERO) and registration number in the Abstract. | Yes | Page 1 (Title Page) - OSF Registration DOI provided |
| **Authors** | | | | |
| Contact | 3a | Provide name, institutional affiliation, and e-mail address of all protocol authors; provide physical mailing address of corresponding author. | Yes | Page 1 (Title Page) |
| Contributions | 3b | Describe contributions of protocol authors and identify the guarantor of the review. | Yes | Page 17 (Authors' contributions) |
| Amendments | 4 | If the protocol represents an amendment of a previously completed or published protocol, identify as such and list changes; otherwise, state plan for documenting important protocol amendments. | N/A | N/A - Initial protocol submission |
| **Support** | | | | |
| Sources | 5a | Indicate sources of financial or other support for the review. | Yes | Page 16 (Funding) |
| Sponsor | 5b | Provide name for the review funder and/or sponsor. | Yes | Page 16 (Funding) |
| Role of sponsor/funder | 5c | Describe roles of funder(s), sponsor(s), and/or institution(s), if any, in developing the protocol. | Yes | Page 16 (Funding) |
| **INTRODUCTION** | | | | |
| Rationale | 6 | Describe the rationale for the review in the context of what is already known. | Yes | Pages 4-8 (Background & Mechanistic paradigms) |
| Objectives | 7 | Provide an explicit statement of the question(s) the review will address with reference to participants, interventions, comparators, and outcomes (PICO). | Yes | Pages 8-9 (Objectives); Pages 9-11 (Eligibility criteria - PCC framework used) |
| **METHODS** | | | | |
| Eligibility criteria | 8 | Specify the study characteristics (e.g., PICO, study design, setting, time frame) and report characteristics to be used as criteria for eligibility for the review. | Yes | Pages 9-11 (Eligibility criteria); Page 26 (Appendix 3: PCC Framework) |
| Information sources | 9 | Describe all intended information sources (e.g., electronic databases, contact with study authors, trial registers, or other grey literature sources) with planned dates of coverage. | Yes | Page 11 (Search strategy) |
| Search strategy | 10 | Present draft of search strategy to be used for at least one electronic database, including planned limits, such that it could be repeated. | Yes | Pages 22-23 (Appendix 1: PubMed Search Strategy) |
| **STUDY RECORDS** | | | | |
| Data management | 11a | Describe the mechanism(s) that will be used to manage records and data throughout the review. | Yes | Pages 11-12 (Study selection) |
| Selection process | 11b | State the process that will be used for selecting studies (e.g., two independent reviewers) through each phase of the review. | Yes | Pages 11-12 (Study selection) |
| Data collection process | 11c | Describe planned method of extracting data from reports (e.g., piloting forms, done independently, in duplicate), any processes for obtaining and confirming data from investigators. | Yes | Pages 12-13 (Data extraction) |
| Data items | 12 | List and define all variables for which data will be sought (e.g., PICO items, funding sources), any pre-planned data assumptions and simplifications. | Yes | Pages 12-13 (Data extraction); Pages 24-25 (Appendix 2: Data Extraction Form) |
| Outcomes and prioritization | 13 | List and define all outcomes for which data will be sought, including prioritization of main and additional outcomes, with rationale. | Yes | Page 10 (Eligibility criteria - Concept); Page 24 (Appendix 2) |
| Risk of bias in individual studies | 14 | Describe anticipated methods for assessing risk of bias of individual studies, including whether this will be done at the outcome or study level, or both; state how this information will be used in data synthesis. | N/A | N/A - This is a Scoping Review. Formal risk of bias assessment is not mandated. |
| **DATA** | | | | |
| Synthesis | 15a | Describe criteria under which study data will be quantitatively synthesized. | N/A | N/A - This is a Scoping Review. Quantitative meta-analysis is not planned. |
|  | 15b | If data are appropriate for quantitative synthesis, describe planned summary measures, methods of handling data, and methods of combining data from studies. | N/A | N/A - This is a Scoping Review. |
|  | 15c | Describe any proposed additional analyses (e.g., sensitivity or subgroup analyses, meta-regression). | Yes | Pages 13-14 (Data synthesis and presentation - Describes Evidence Matrix and stratification) |
|  | 15d | If quantitative synthesis is not appropriate, describe the type of summary planned. | Yes | Pages 13-14 (Data synthesis and presentation) |
| Meta-bias(es) | 16 | Specify any planned assessment of meta-bias(es) (e.g., publication bias across studies, selective reporting within studies). | N/A | N/A - This is a Scoping Review. |
| Confidence in cumulative evidence | 17 | Describe how the strength of the body of evidence will be assessed (e.g., GRADE). | N/A | N/A - This is a Scoping Review. |
