# Supplementary material for: Autonomic nervous system modulation by G protein-biased mu-opioid receptor agonists: A translational scoping review protocol
Source: PLoS One. 2026 May 15;21(5):e0349596. doi: 10.1371/journal.pone.0349596 (PMC13178854; doi:10.1371/journal.pone.0349596)
Supplement: S2 Checklist — (DOCX) [file pone.0349596.s002.docx]

# **S2 Checklist. PRISMA-ScR checklist**

*Preferred Reporting Items for Systematic reviews and Meta-Analyses extension for Scoping Reviews*

**Review title:** Autonomic nervous system modulation by G protein-biased mu-opioid receptor agonists: a translational scoping review protocol

| **SECTION** | **ITEM** | **PRISMA-ScR CHECKLIST ITEM** | **LOCATION** |
| --- | --- | --- | --- |
| **TITLE** | | | |
|  | 1. Title | Identify the report as a scoping review. | Title page |
| **ABSTRACT** | | | |
|  | 2. Structured summary | Provide a structured summary including background, objectives, eligibility criteria, sources of evidence, charting methods, results, and conclusions. | Abstract |
| **INTRODUCTION** | | | |
|  | 3. Rationale | Describe the rationale for the review in the context of existing knowledge. Explain why the review questions lend themselves to a scoping review approach. | Background section |
|  | 4. Objectives | State the questions and objectives being addressed with reference to key elements (population, concepts, context). | Objectives section |
| **METHODS** | | | |
|  | 5. Protocol and registration | Indicate whether a protocol exists; provide registration information if available. | Protocol design; OSF registration |
|  | 6. Eligibility criteria | Specify characteristics of evidence sources used as eligibility criteria and provide rationale. | Eligibility criteria |
|  | 7. Information sources | Describe all information sources and the date of the most recent search. | Search strategy |
|  | 8. Search | Present the full electronic search strategy for at least one database. | Additional file 1 |
|  | 9. Selection of sources of evidence | State the process for selecting sources of evidence. | Study selection |
|  | 10. Data charting process | Describe methods of charting data from included sources. | Data extraction |
|  | 11. Data items | List and define all variables for which data were sought. | Data extraction; Additional file 2 |
|  | 12. Critical appraisal of individual sources | If done, provide rationale and describe methods used. | Not applicable |
|  | 13. Synthesis of results | Describe methods of handling and summarizing charted data. | Data synthesis and presentation |
| **RESULTS** | | | |
|  | 14. Selection of sources of evidence | Provide numbers of sources screened, assessed for eligibility, and included. | To be reported |
|  | 15. Characteristics of sources of evidence | Present characteristics for which data were charted. | To be reported |
|  | 16. Critical appraisal within sources | Present critical appraisal data if conducted. | Not applicable |
|  | 17. Results of individual sources | Present relevant charted data for each included source. | To be reported |
|  | 18. Synthesis of results | Summarize charting results as they relate to review questions. | To be reported |
| **DISCUSSION** | | | |
|  | 19. Summary of evidence | Summarize main results and consider relevance to key groups. | Discussion |
|  | 20. Limitations | Discuss limitations of the scoping review process. | Limitations |
|  | 21. Conclusions | Provide interpretation of results with potential implications and next steps. | Discussion; Ethics and dissemination |
| **FUNDING** | | | |
|  | 22. Funding | Describe sources of funding and the role of funders. | Funding |

***Source:*** *Tricco AC, Lillie E, Zarin W, et al. PRISMA Extension for Scoping Reviews (PRISMA-ScR): Checklist and Explanation. Ann Intern Med. 2018;169(7):467-473.*

***Note:*** *Items 14-18 (Results section) will be completed in the final review manuscript. This is a protocol publication.*
