# Supplementary material for: Autonomic nervous system modulation by G protein-biased mu-opioid receptor agonists: A translational scoping review protocol
Source: PLoS One. 2026 May 15;21(5):e0349596. doi: 10.1371/journal.pone.0349596 (PMC13178854; doi:10.1371/journal.pone.0349596)
Supplement: S1 Appendix — (DOCX) [file pone.0349596.s003.docx]

# **S1 Appendix. Search strategy**

*Autonomic nervous system modulation by G protein-biased mu-opioid receptor agonists: a translational scoping review*

| **Database** | PubMed (MEDLINE) |
| --- | --- |
| **Date of search** | [To be completed upon execution] |
| **Coverage** | Database inception through March 2026 |

## **Concept 1: Biased/Low-Efficacy MOR Agonists**

**#1** "oliceridine"[tiab] OR "TRV130"[tiab] OR "TRV-130"[tiab] OR "Olinvyk"[tiab]

**#2** "tegileridine"[tiab] OR "SHR8554"[tiab] OR "SHR-8554"[tiab] OR "Aisute"[tiab]

**#3** "PZM21"[tiab] OR "SR-17018"[tiab] OR "TRV734"[tiab]

**#4** #1 OR #2 OR #3

**#5** "biased agonist"[tiab] OR "biased agonism"[tiab] OR "G protein-biased"[tiab]

**#6** "functional selectivity"[tiab] OR "functionally selective"[tiab]

**#7** ("low efficacy"[tiab] OR "partial agonist"[tiab]) AND "opioid"[tiab]

**#8** #5 OR #6 OR #7

## **Concept 2: Mu-Opioid Receptor**

**#9** "Receptors, Opioid, mu"[MeSH] OR "mu-opioid"[tiab] OR "mu opioid"[tiab]

**#10** "MOR"[tiab] OR "OPRM1"[tiab]

**#11** #9 OR #10

**#12** (#4 OR #8) AND #11

## **Concept 3: Autonomic/Cardiovascular Outcomes**

**#13** "Heart Rate"[MeSH] OR "Blood Pressure"[MeSH] OR "Autonomic Nervous System"[MeSH]

**#14** "heart rate variability"[tiab] OR "HRV"[tiab] OR "RMSSD"[tiab] OR "SDNN"[tiab]

**#15** "cardiovascular"[tiab] OR "hemodynamic"[tiab]

**#16** "bradycardia"[tiab] OR "hypotension"[tiab] OR "QT"[tiab] OR "QTc"[tiab]

**#17** "autonomic"[tiab] OR "vagal"[tiab] OR "sympathetic"[tiab]

**#18** "baroreflex"[tiab] OR "catecholamine"[tiab]

**#19** #13 OR #14 OR #15 OR #16 OR #17 OR #18

## **Combined Search**

**#20** #12 AND #19

## **Mechanistic Context**

**#21** "beta-arrestin"[tiab] OR "ARRB2"[tiab]

**#22** #21 AND #19 AND ("opioid"[tiab] OR "receptor"[tiab])

## **Final Search**

**#23** #20 OR #22

## **Other Databases**

We will adapt this search strategy for:

Embase via Ovid

Web of Science Core Collection

Cochrane Central Register of Controlled Trials

CNKI, Wanfang Data, and SinoMed (no language restrictions)

## **Grey Literature and Regulatory Sources**

FDA Drugs@FDA: NDA 210730 (oliceridine)

CDE/NMPA database (tegileridine)

ClinicalTrials.gov and ChiCTR

FAERS for post-marketing cardiovascular signals
