# Supplementary material for: Autonomic nervous system modulation by G protein-biased mu-opioid receptor agonists: A translational scoping review protocol
Source: PLoS One. 2026 May 15;21(5):e0349596. doi: 10.1371/journal.pone.0349596 (PMC13178854; doi:10.1371/journal.pone.0349596)
Supplement: S2 Appendix — (DOCX) [file pone.0349596.s004.docx]

# **S2 Appendix. Data extraction form**

*Autonomic nervous system modulation by G protein-biased mu-opioid receptor agonists: a translational scoping review*

| **Category** | **Data Items** |
| --- | --- |
| **Study identification** | First author, publication year, country, journal name, DOI, funding sources, declared conflicts of interest |
| **Study design** | Evidence level (in vitro / animal / phase I-IV / observational / regulatory document) |
| **Drug/intervention** | Drug name, dose(s), administration route, treatment duration; comparator(s) when applicable |
| **Bias characterization** | Bias source (ligand-driven vs. receptor-engineered); Bias factor (numeric value or qualitative rating: high/moderate/low); reference ligand for bias calculation (morphine, DAMGO, etc.); intrinsic efficacy (Emax) when reported |
| **Population/model** | In vitro studies: cell line, receptor type. Animal studies: species, strain, model type, anesthetic state (conscious or anesthetized with agent specified). Clinical studies: sample size, patient characteristics, key demographics |
| **Measurement timepoints** | Baseline / Acute (within 2h) / Early recovery (2-24h) / Late recovery (beyond 24h) |
| **Primary autonomic outcomes** | HRV indices (specify: RMSSD, SDNN, LF, HF, LF/HF ratio, etc.); baroreflex sensitivity; pupillometry; plasma catecholamines; direct nerve recordings |
| **Cardiovascular safety** | Heart rate, blood pressure, QT/QTc interval, arrhythmia occurrence, reported cardiovascular adverse events |
| **Key findings** | Main results with effect sizes when available; dose-response data if reported; statistical comparisons between groups |
| **Paradigm alignment** | Aligns with: Bias Preservation / Efficacy Ceiling / Null / Cannot determine |
| **Limitations** | Limitations noted by study authors; methodological concerns identified during extraction |

***Notes for Extractors***

*One reviewer (YZ, QC, or YyZ) will extract data; a second reviewer (HC or QZ) will check all entries for accuracy.*

*Record the reference ligand used when bias factors are reported, as different reference compounds yield different values.*

*For animal experiments, always note whether subjects were conscious or under anesthesia, and name the anesthetic agent.*

*Use the timepoint categories consistently to allow comparison across studies.*

*If data are missing, contact corresponding authors (up to two attempts per study).*

*Select "Cannot determine" for hypothesis relevance when evidence is inconclusive or contradictory.*
