# Supplementary material for: Autonomic nervous system modulation by G protein-biased mu-opioid receptor agonists: A translational scoping review protocol
Source: PLoS One. 2026 May 15;21(5):e0349596. doi: 10.1371/journal.pone.0349596 (PMC13178854; doi:10.1371/journal.pone.0349596)
Supplement: S3 Appendix — (DOCX) [file pone.0349596.s005.docx]

# **S3 Appendix. PCC framework summary**

*Autonomic nervous system modulation by G protein-biased mu-opioid receptor agonists: a translational scoping review*

The table below outlines the Population, Concept, and Context elements that define study eligibility for this scoping review.

| **Element** | **Inclusion** | **Exclusion** |
| --- | --- | --- |
| **Population/ Exposure** | G protein-biased or low-efficacy MOR agonists, specifically oliceridine (TRV130, Olinvyk), tegileridine (SHR8554, Aisute), PZM21, SR-17018, TRV734, and newer investigational compounds. We will also include studies using beta-arrestin-2 knockout mice or phosphorylation-deficient MOR mutant models. | Studies examining only conventional opioids without any biased agonist comparator |
| **Concept** | Primary outcomes: autonomic function measures such as HRV parameters (RMSSD, SDNN, LF, HF, LF/HF ratio), baroreflex sensitivity, pupillometry, catecholamine concentrations, and direct nerve activity recordings. Secondary outcomes: cardiovascular safety data including heart rate, blood pressure, QT/QTc interval, and cardiovascular adverse events. | Studies reporting only non-cardiovascular outcomes with no autonomic data whatsoever |
| **Context** | All study settings will be considered: in vitro receptor signaling assays, animal experiments (with stratification by conscious versus anesthetized state), healthy volunteer pharmacokinetic and thorough QT studies, clinical trials in surgical or medical patients, regulatory submission data from FDA and NMPA, and post-marketing surveillance reports. | No context restrictions apply |

***Source Types***

*We will include original research articles (in vitro, animal, clinical trials of any phase, observational studies) and regulatory documents containing cardiovascular safety information. Reviews will be excluded from the primary analysis, though we will screen their reference lists for additional primary studies. Conference abstracts, case reports (unless they describe novel cardiovascular adverse events), There will be no language restrictions. We will systematically search major Chinese databases (CNKI, Wanfang Data, and SinoMed) to ensure comprehensive capture of relevant data.*

***Timepoint Categorization***

*To enable cross-study comparison, we will group measurement timepoints into four categories:*

***Baseline:*** *measurements obtained before drug administration*

***Acute phase:*** *during infusion or within 2 hours of dosing*

***Early recovery:*** *2 to 24 hours post-administration*

***Late recovery:*** *beyond 24 hours*
