# Supplementary material for: Autonomic nervous system modulation by G protein-biased mu-opioid receptor agonists: A translational scoping review protocol
Source: PLoS One. 2026 May 15;21(5):e0349596. doi: 10.1371/journal.pone.0349596 (PMC13178854; doi:10.1371/journal.pone.0349596)
Supplement: Attachment — Submitted filename: renamed_cd645.docx [file pone.0349596.s006.docx]

**Reviewer’s Comment**

This manuscript is solid but over-claimed preclinical study. The central idea of using umbelliferone to potentiate doxorubicin efficacy while mitigating hepatorenal toxicity is plausible and clinically relevant, but the manuscript currently overstates mechanistic certainty, under-controls key experiments, and blurs correlation with causation, particularly regarding FOXO3a-dependent autophagy and oxidative stress. Below are what should be corrected before the manuscript can be accepted for publication.

1. The mechanistic role of FOXO3a is inferred from expression changes rather than confirmed through functional inhibition or gene-silencing experiments.
2. Nuclear localization and transcriptional activity of FOXO3a were not assessed, limiting confidence in its regulatory involvement.
3. Autophagy assessment relied on static markers (LC3, Beclin-1) without evaluation of autophagic flux or lysosomal turnover.
4. The absence of autophagy inhibition or flux assays prevents distinction between increased autophagy initiation and impaired degradation.
5. The interaction between umbelliferone and doxorubicin was not evaluated using pharmacological synergy models, restricting interpretation to additive effects.
6. Dose response interaction analysis was not performed to define the optimal combination or therapeutic window.
7. Potential effects of umbelliferone on doxorubicin pharmacokinetics or tissue distribution were not examined.
8. Hepatorenal protection was evaluated primarily using biochemical and histopathological markers without complementary functional endpoints.
9. Renal filtration capacity and broader hepatic metabolic function were not directly assessed.
10. The study was conducted using a single experimental model and dosing regimen, limiting generalizability across cancer types and physiological contexts.
11. Translational relevance remains constrained by the lack of long-term outcome and survival analyses.
