# Supplementary material for: Autonomic nervous system modulation by G protein-biased mu-opioid receptor agonists: A translational scoping review protocol
Source: PLoS One. 2026 May 15;21(5):e0349596. doi: 10.1371/journal.pone.0349596 (PMC13178854; doi:10.1371/journal.pone.0349596)
Supplement: Attachment — Submitted filename: Response to Reviewers.docx [file pone.0349596.s007.docx]

**Response to Reviewers**

**Dear Dr. Yusuf Oloruntoyin Ayipo,**

Thank you for giving us the opportunity to submit a revised draft of our manuscript. We deeply appreciate the time and effort you and the five reviewers have dedicated to evaluating our protocol. The reviewers’ comments, particularly regarding the inclusion of in vitro studies, the distinction between ligand-driven and receptor-engineered bias, and the removal of language restrictions, were incredibly insightful. Incorporating these suggestions has significantly improved the methodological rigor and clarity of our protocol.

We have carefully addressed every comment raised by the reviewers. Please find our point-by-point responses below. The corresponding changes have been highlighted in the "Revised Manuscript with Track Changes" file.

# Response to Journal Requirements

## 1. PRISMA-P checklist requirement.

Response: Thank you for this guidance. We have completed the PRISMA-P 2015 checklist and uploaded it as a separate Supporting Information file (S1 Checklist). Since this is a scoping review protocol, we have marked items exclusively pertaining to systematic reviews/meta-analyses (e.g., risk of bias assessment, meta-biases) as "N/A", while providing the relevant page numbers for all other items.

## 2. Removal of funding information from the manuscript.

Response: We have removed the "Funding" section from the main text of the manuscript. The funding information is now only provided in the online submission form.

## 3. ORCID iD validation.

Response: The corresponding author (Yukai Zhou) has successfully linked and validated his ORCID iD in the Editorial Manager system.

## 4. Captions for Supporting Information files.

Response: We have added a "Supporting information" section at the end of the manuscript (after the References) with clear captions for all supplementary files, and we have updated all in-text citations accordingly (e.g., changing "Appendix 1" to "S2 Appendix").

# Response to Reviewer #1

**Comment 1.1 & 1.2:** Lack of deeper methodological rigor and no clear plan for handling heterogeneity, bias assessment, or data quality weighting.
**Response:** We completely agree with your concern. Initially, we omitted a formal quality assessment because it is optional in scoping reviews. However, given the mechanistic controversies in this field, we realize that assessing study quality is crucial. We have now added a "Critical appraisal" section. We will use the JBI Critical Appraisal Checklists for clinical studies and the SYRCLE risk of bias tool for animal studies. For in vitro studies, we will assess the methodological rigor of bias factor calculations.
**Action taken:** A new "Critical appraisal" section has been added to the Methods (Page 13).

**Comment 1.3:** Including preclinical, clinical, mechanistic, and regulatory data together introduces substantial heterogeneity.
**Response:** Thank you for pointing this out. We acknowledge the high heterogeneity. To prevent meaningless pooling of data, we will strictly stratify our synthesis. We have updated the "Data synthesis and presentation" section to clarify that data will be synthesized within their specific evidence levels (e.g., in vitro, animal, human) rather than across them.
**Action taken:** Clarified in the "Data synthesis and presentation" section (Page 14).

**Comment 1.4:** No alternative structured approach (e.g., semi-quantitative synthesis, vote counting, or effect direction analysis) since meta-analysis is not planned.
**Response:** This is a very helpful suggestion. We have added a plan to use an "effect direction plot" alongside our Evidence Matrix. This will visually represent the direction of autonomic effects (e.g., HRV preserved, HRV reduced, or conflicting) across different drug doses and study types.
**Action taken:** Added to the "Data synthesis and presentation" section (Page 15).

**Comment 1.5:** The three paradigms (bias preservation, efficacy ceiling, null) are not operationalized; there are no predefined criteria.
**Response:** We apologize for the previous vagueness. We have now provided explicit, predefined criteria for how we will assign the extracted evidence to each of the three mechanistic paradigms.
**Action taken:** Detailed operational definitions for Paradigm 1, 2, and 3 have been added to the "Data synthesis and presentation" section (Pages 14-15).

**Comment 1.6:** Exclusion of non-English studies introduces bias and may omit critical data (especially for tegileridine).
**Response:** We completely agree. As clinical researchers from China, we initially limited the search to English to match standard international review practices, but we realize this would miss crucial data for tegileridine. We have removed the language restriction and officially added major Chinese databases (CNKI, Wanfang Data, and SinoMed) to our search strategy.
**Action taken:** Updated in the "Types of sources" (Page 11), "Limitations" (Page 16), and the search strategy appendices.

**Comment 1.7:** The research question is sufficiently focused that a systematic review could be more appropriate. Why a scoping review?
**Response:** Thank you for this methodological question. We chose a scoping review because we are mapping a highly heterogeneous body of literature that spans the entire translational continuum (from in vitro cell assays to in vivo animal models and human trials). The varying operational definitions of "biased agonism" and the exploratory nature of linking these to autonomic outcomes preclude the narrow PICO framework required for a systematic review. We have added this justification to the manuscript.
**Action taken:** Added a rationale paragraph under "Objectives" (Page 9).

**Comment 1.8:** ANS focus is interesting but not strongly positioned as a significant gap.
**Response:** We have strengthened the Introduction to better highlight the clinical and mechanistic gap. Specifically, we introduced the "GIRK Paradox" (G-protein-gated inwardly rectifying potassium channels), explaining that since vagal modulation is largely G-protein dependent, it remains highly uncertain whether reducing β-arrestin alone can preserve autonomic homeostasis.
**Action taken:** Added mechanistic details to the "The autonomic gap" section in the Introduction (Page 6).

**Comment 1.9:** The search strategy is mentioned but not fully detailed in the main text.
**Response:** We have expanded the description of the search strategy in the main text to clearly show the three-block conceptual framework we used.
**Action taken:** Updated the "Search strategy" section (Page 11).

**Comment 1.10:** Distinction between autonomic indices and cardiovascular outcomes is acknowledged but not clearly resolved.
**Response:** We have refined our definitions to draw a strict conceptual distinction. We now explicitly state that primary autonomic indices (e.g., HRV, pupillometry) directly reflect neural tone, while secondary cardiovascular outcomes (e.g., blood pressure, QT interval) are composite measures influenced by both autonomic tone and direct cardiac effects.
**Action taken:** Clarified in the "Concept" section of the Eligibility criteria (Page 10).

**Comment 1.11:** Protocol does not clearly state how contradictory findings will be synthesized or interpreted.
**Response:** We have clarified that our newly added quality assessment (critical appraisal) and the effect direction plot will be used specifically to explore whether contradictory findings stem from methodological flaws or unmeasured confounders (such as different anesthetic agents used in animal models).
**Action taken:** Addressed in the "Critical appraisal" and "Data synthesis" sections (Pages 13-14).

**Comment 1.12:** The proposed “Evidence Matrix” lacks detail on how it will be constructed.
**Response:** We have provided specific details on the axes of the Evidence Matrix: Dimension 1 (Intervention Type), Dimension 2 (Evidence Level), and Dimension 3 (Timepoint).
**Action taken:** Detailed in the "Data synthesis and presentation" section (Pages 13-14).

**Comment 1.13:** Important confounders (dose, PK, patient condition, anesthesia effects) are not formally incorporated into analysis strategy.
**Response:** We agree that these confounders are critical. We have added a specific plan to synthesize data using stratified analyses. For example, clinical data will be stratified by patient condition and concurrent anesthesia, and preclinical data will be strictly separated by anesthetic state (conscious vs. anesthetized).
**Action taken:** Added to the "Data synthesis and presentation" section (Page 14).

# Response to Reviewer #2

**Comment 2.1:** Clarify why opt to select scoping review rather than systematic review.
**Response:** Thank you for the comment. As also requested by Reviewer 1, we have added a clear justification. The inclusion of in vitro assays, animal models, and clinical trials creates a translational spectrum that is too broad for a traditional systematic review. A scoping review allows us to map these diverse evidence types to identify gaps (especially for tegileridine) before narrower systematic reviews can be conducted.
**Action taken:** Added to the "Objectives" section (Page 9).

**Comment 2.2:** Make clear justification how the GIRK paradox and bias factor heterogeneity will be handled. Figure 1 caption could be shortened.
**Response:** We greatly appreciate this advice. We have significantly shortened the caption for Figure 1 and moved the detailed explanation of the "GIRK Paradox" into the Introduction text. Furthermore, to handle bias factor heterogeneity, we will explicitly extract the calculation methods and reference ligands (e.g., DAMGO vs. morphine) used in each study.
**Action taken:** Figure 1 caption shortened (Page 8). GIRK paradox moved to Introduction (Page 6). Bias factor extraction detailed in "Data extraction" (Page 13).

**Comment 2.3:** Address how the English-language restriction may affect the depth and completeness of evidence for tegileridine.
**Response:** We agree this was a major limitation. We have completely removed the English-language restriction and added major Chinese databases (CNKI, Wanfang Data, SinoMed) to our search strategy to ensure we capture all available data for tegileridine.
**Action taken:** Updated in "Types of sources" (Page 11) and "Limitations" (Page 16).

# Response to Reviewer #3

**Comment 3.1:** I still question the decision to only include English language articles given the presence of these drugs in China.
**Response:** We sincerely thank the reviewer for pointing out this critical flaw. We have entirely removed the language restriction. We will systematically search Chinese databases (CNKI, Wanfang Data, and SinoMed) to ensure no tegileridine data is missed.
**Action taken:** Search strategy and limitations updated accordingly (Pages 11, 16).

# Response to Reviewer #4

**Comment 4.1:** Clearer distinction needed between ligand-driven (pharmacological) bias and receptor-engineered (genetic) bias (e.g., Kliewer et al. 2019). State how they will be interpreted and analyzed separately.
**Response:** We are very grateful for this expert insight. We completely agree that findings from phosphorylation-deficient mutant mice cannot be directly translated to the clinical effects of biased drugs. We have added a paragraph in the Introduction to explicitly distinguish between ligand-driven and receptor-engineered bias. Furthermore, we have updated our data extraction form to capture the "bias source", and we will stratify these two distinct experimental approaches in our Evidence Matrix.
**Action taken:** Added distinction in Introduction (Page 6). Updated "Data extraction" (Page 13) and "Data synthesis" (Page 14).

**Comment 4.2:** Grouping “G protein-biased or low-efficacy MOR agonists” conflates two mechanistically distinct pharmacological concepts. Clarify how these classes will be defined and distinguished.
**Response:** You are absolutely correct. Bias and intrinsic efficacy are fundamentally different concepts, and combining them was a conceptual error in our previous draft. To resolve this, we will explicitly extract intrinsic efficacy values (e.g., Emax for G-protein activation) and bias factors. During data synthesis, we will use these extracted values to stratify the drugs into "biased agonists" versus "low-efficacy/partial agonists" to see if their autonomic profiles differ.
**Action taken:** Clarification added to the "Data extraction" section (Page 13).

**Comment 4.3:** Limited emphasis on in vitro or mechanistic studies may constrain the ability to properly contextualize translational findings.
**Response:** We appreciate this important feedback. As clinical researchers, we initially excluded in vitro studies to keep the review manageable. However, we agree that without in vitro signaling data, we cannot accurately define "bias" or "efficacy." We have now officially included in vitro cellular assays in our eligibility criteria.
**Action taken:** In vitro studies added to "Eligibility criteria" (Pages 9, 10, 11).

**Comment 4.4:** Clarification is needed on how heterogeneous evidence sources (e.g., controlled trials, PK studies, post-marketing) will be categorized and synthesized.
**Response:** We have refined our "Data synthesis and presentation" section. We will use an Evidence Matrix to strictly separate the data by Evidence Level (Dimension 2: in vitro, preclinical in vivo, healthy volunteers, patient populations). We will not pool data across these different study designs.
**Action taken:** Detailed in the "Data synthesis and presentation" section (Page 14).

# Response to Reviewer #5

**Comment 5.1:** Conceptual clarity: Relating heterogeneous evidence to mechanistic paradigms may overreach a scoping review. State that such interpretations are hypothesis-generating rather than definitive.
**Response:** We agree with your assessment. A scoping review cannot definitively prove a pharmacological mechanism. To avoid overreaching, we have changed the term "competing hypotheses" to "mechanistic paradigms". We have also added an explicit statement acknowledging that assigning heterogeneous evidence to these paradigms is purely exploratory and hypothesis-generating.
**Action taken:** Terminology updated throughout the manuscript. Explicit disclaimer added at the end of the "Data synthesis and presentation" section (Page 15).

**Comment 5.2:** Definition of biased agonism: The inclusion criteria combine biased agonists with low-efficacy/partial agonists without a clear operational definition.
**Response:** As also suggested by Reviewer 4, we have corrected this conflation. We will extract specific bias factors and intrinsic efficacy measures (Emax) to operationally distinguish true biased agonists from low-efficacy partial agonists during the data synthesis phase.
**Action taken:** Added to the "Data extraction" section (Page 13).

**Comment 5.3:** Synthesis approach: The protocol should better distinguish between different levels of evidence (molecular, animal, clinical) to avoid overinterpretation.
**Response:** We have updated the Evidence Matrix description to ensure that molecular, preclinical, and clinical data are strictly stratified and synthesized within their own evidence levels.
**Action taken:** Clarified in the "Data synthesis and presentation" section (Page 14).

**Comment 5.4:** Language restriction bias: Limiting to English-language studies may exclude relevant data, especially for tegileridine.
**Response:** We have removed the English-language restriction and included Chinese databases (CNKI, Wanfang, SinoMed) in our search strategy.
**Action taken:** Updated in "Types of sources" (Page 11).

**Comment 5.5:** Quality assessment: The absence of any plan for study quality or risk-of-bias assessment should be justified.
**Response:** We agree that given the controversies in this field, quality assessment is necessary. We have added a "Critical appraisal" section, utilizing JBI tools for clinical studies and SYRCLE for animal studies, to inform our narrative synthesis.
**Action taken:** Added "Critical appraisal" section (Page 13).

Once again, we thank the Academic Editor and the Reviewers for their highly constructive comments, which have undoubtedly elevated the quality of our protocol. We hope the revised manuscript is now suitable for publication in PLOS ONE.

Sincerely,

Yukai Zhou, MD
Corresponding Author
On behalf of all co-authors
